# The Relative Expression of Mig6 and EGFR Is Associated with Resistance to EGFR Kinase Inhibitors

**DOI:** 10.1371/journal.pone.0068966

**Published:** 2013-07-31

**Authors:** Xiaofei Chang, Eugene Izumchenko, Luisa M. Solis, Myoung Sook Kim, Aditi Chatterjee, Shizhang Ling, Constance L. Monitto, Paul M. Harari, Manuel Hidalgo, Steve N. Goodman, Ignacio I. Wistuba, Atul Bedi, David Sidransky

**Affiliations:** 1 Department of Otolaryngology-Head and Neck Surgery, Division of Head and Neck Cancer Research, Johns Hopkins University, Baltimore, Maryland, United States of America; 2 Departments of Pathology and Thoracic/Head and Neck Medical Oncology, University of Texas MD Anderson, Houston, Texas, United States of America; 3 Department of Pathology, Johns Hopkins University, Baltimore, Maryland, United States of America; 4 Department of Anesthesiology and Critical Care Medicine, Johns Hopkins University, Baltimore, Maryland, United States of America; 5 Department of Human Oncology, University of Wisconsin, School of Medicine and Public Health, Madison, Wisconsin, United States of America; 6 Sidney Kimmel Comprehensive Cancer Center, School of Medicine, Johns Hopkins University, Baltimore, Maryland, United States of America; 7 Centro Intregral Oncologico Clara Campal, Madrid, Spain; 8 Department of Medicine, Stanford University School of Medicine, Stanford, California, United States of America; University of Nebraska Medical Center, United States of America

## Abstract

The sensitivity of only a few tumors to anti-epidermal growth factor receptor EGFR tyrosine kinase inhibitors (TKIs) can be explained by the presence of EGFR tyrosine kinase (TK) domain mutations. In addition, such mutations were rarely found in tumor types other than lung, such as pancreatic and head and neck cancer. In this study we sought to elucidate mechanisms of resistance to EGFR-targeted therapies in tumors that do not harbor TK sensitizing mutations in order to identify markers capable of guiding the decision to incorporate these drugs into chemotherapeutic regimens. Here we show that EGFR activity was markedly decreased during the evolution of resistance to the EGFR tyrosine kinase inhibitor (TKI) erlotinib, with a concomitant increase of mitogen-inducible gene 6 (Mig6), a negative regulator of EGFR through the upregulation of the PI3K-AKT pathway. EGFR activity, which was more accurately predicted by the ratio of Mig6/EGFR, highly correlated with erlotinib sensitivity in panels of cancer cell lines of different tissue origins. Blinded testing and analysis in a prospectively followed cohort of lung cancer patients treated with gefitinib alone demonstrated higher response rates and a marked increased in progression free survival for patients with a low Mig6/EGFR ratio (approximately 100 days, *P* = 0.01).

## Introduction

Selective small molecule tyrosine kinase inhibitors (TKIs) of EGFR, such as gefitinib and erlotinib, were among the first targeted therapies developed for cancer. Some of these inhibitors have demonstrated benefit in select clinical settings, however, primary as well as acquired drug resistance eventually arises in most, if not all, treated patients [1], [2], [3]. While primary somatic mutations in the tyrosine kinase domain of EGFR render tumors more sensitive to gefitinib and/or erlotinib [1], [4], and secondary mutations are associated with acquired drug resistance [3], [5], these genetic alterations are present in only a minority of patients who partially respond to treatment and are rare in tumors other than NSCLCs [2], [6], [7], [8]. In order to be able to provide treatment selectively to those patients who do not harbor EGFR mutations but will nonetheless respond to TKIs, there is an urgent need to define the precise molecular mechanisms underlying resistance to EGFR-targeted TKIs, and to identify specific biomarkers capable of predicting therapeutic response.

Efforts have been made to correlate EGFR protein levels with the response to anti-EGFR therapy, however, the relationship between the two has been surprisingly poor [2], [8], [9], [10]. A fact that is commonly overlooked is that EGFR expression may be uncoupled from its activity via negative feedback regulators of EGFR family receptor tyrosine kinases (RTKs). Among these negative regulators, the multiadaptor protein mitogen-inducible gene 6 (Mig6, also known as RALT, ERRFI1 or Gene 33), plays an important role in signal attenuation of the EGFR network by blocking the formation of the activating dimer interface through interaction with the kinase domains of EGFR and ERBB2 [11], [12], [13], [14]. Mig6 knockout (*Errfi1^−/−^*) mice exhibit hyperactivation of endogenous EGFR, resulting in hyperproliferation and impaired differentiation of epidermal keratinocytes. In addition, carcinogen-induced tumors in *Errfi1^−/−^* mice are unusually sensitive to the EGFR TKI gefitinib [15].

In the current study, we observed Mig6 upregulation in acquired erlotinib resistant clone from head and neck cancer cell line. Subsequently, we identified the relative expression of Mig6 and EGFR as a marker of *de novo* responsiveness to erlotinib in a panel of cancer cell lines, and a unique collection of early passage human lung and pancreas tumors xenografts. Tumor responsiveness to erlotinib could be better predicted in some tissue types by measuring expression levels of both EGFR and Mig6 than by measuring expression levels of either protein alone. This finding was further supported by blinded testing of Mig6 and EGFR expression in samples from a small prospective study of patients treated with gefitinib. Taken together these studies highlight the importance of negative cellular regulators of EGFR in predicting sensitivity to TKIs and identify the potential clinical utility of these proteins as predictive biomarkers.

## Results

### Acquired resistance to erlotinib is associated with upregulation of Mig6 and decreased EGFR activity

**Figure 1:**
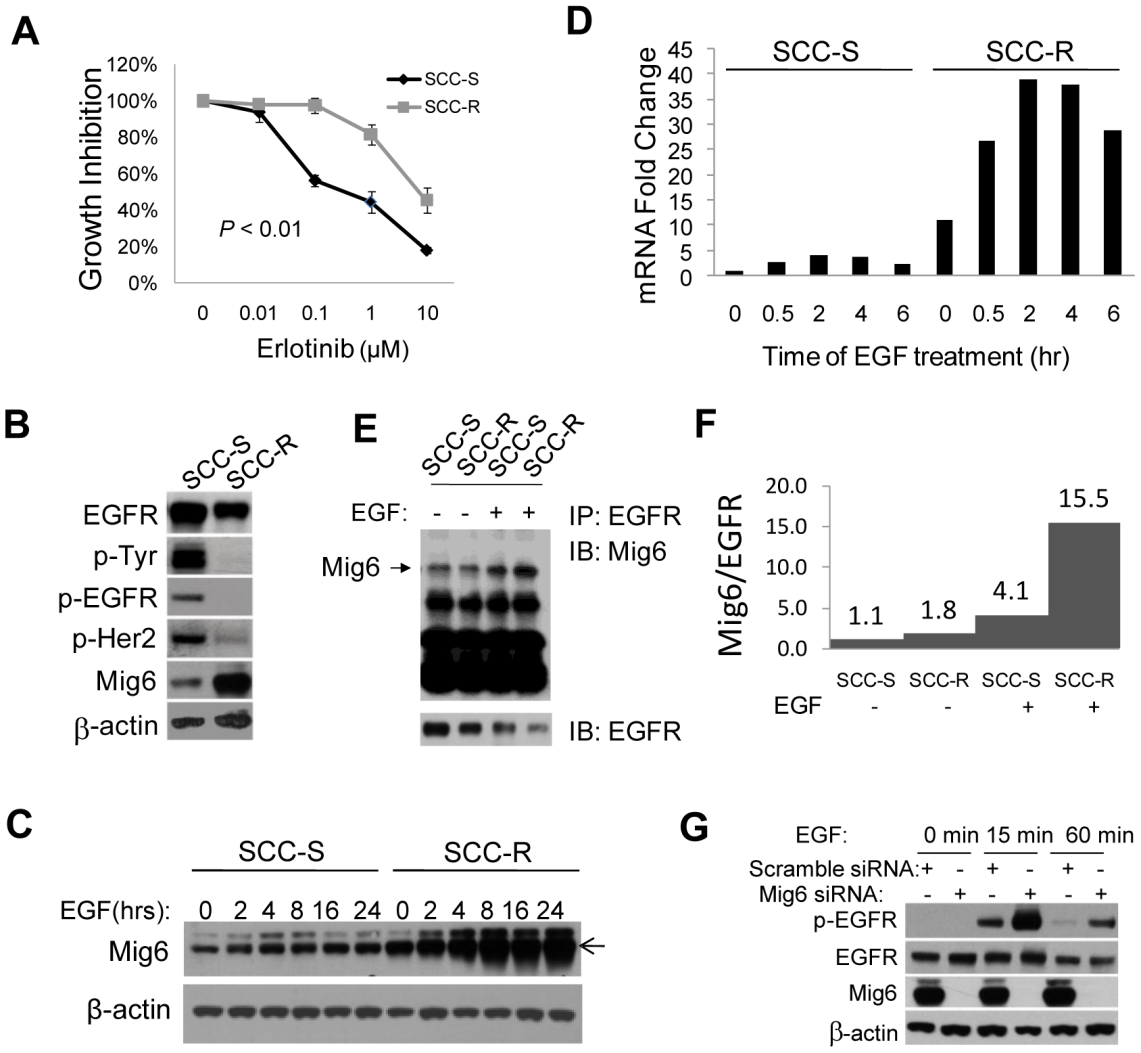
Mig6 is upregulated in an erlotinib resistant cell line which suppresses EGFR phosphorylation. **A**) Erlotinib-sensitive (SCC-S) and -resistant (SCC-R) cells were treated with erlotinib and cell viability was assayed. Values were set at 100% for untreated controls. **B**) Immunoblot analysis of protein expression in SCC-S and -SCC-R cell lines. **C**) SCC-S and SCC-R cells were treated with EGF at the indicated times and Mig6 protein expression was analyzed. **D**) Mig6 mRNA expression was examined by real-time quantitative PCR after EGF treatment at the indicated times. Mig6 mRNA expression was normalized to GAPDH expression. **E**) SCC-S and SCC-R cells were serum-stripped and stimulated with EGF for 60 min. Immunoprecipitation (IP) was performed against EGFR, followed by immunoblotting against Mig6 and EGFR. **F**) Densitometric quantification of Mig6 and EGFR. Data are presented as the ratio of Mig6/EGFR to indicate how many Mig6 molecules are associated with each EGFR molecule. All ratios are presented in relative arbitrary values. **G**) SCC-R cells were transfected with either scrambled siRNA or siRNA targeting Mig6 for 48 hrs. Cells were stripped in serum free medium overnight and stimulated with EGF for 15 or 60 min.

Erlotinib-resistant (SCC-R) and erlotinib-sensitive (SCC-S) isogenic cell lines were generated via chronic exposure of human head and neck squamous cell carcinoma UM-SCC1 cells to either erlotinib or DMSO (vehicle control). The IC_50_ of SCC-R cells was >10 times higher than that seen with SCC-S cells (Figure 1A). Comparing the expression and basal activity of EGFR in SCC-S and SCC-R cell lines we found that the level of phosphorylated EGFR was markedly and disproportionally decreased in SCC-R cells (Figure 1B). This apparent uncoupling of EGFR protein expression and activity in resistant cells was associated with a relatively higher expression of the endogenous *ERBB* family negative regulator, Mig6 (Figure 1B). While treatment with EGF induced a rapid, sustained increase in Mig6 in both cell lines, Mig6 expression remained markedly higher in SCC-R cells as compared to SCC-S cells (Figure 1C and 1D). In addition, more Mig6 was found to be associated with EGFR in SCC-R cells, especially after EGF induction (Figure 1E). Densitometric quantification showed an almost four-fold increase in the level of EGFR engaged by Mig6 in SCC-R cells after ligand stimulation as compared to SCC-S cells (Figure 1F), indicating that overexpressed Mig6 present in SCC-R cells was functionally active. Mig6 knockdown in SCC-R cells resulted in an increase of EGFR phosphorylation in response to treatment with EGF (Figure 1G).

### Mig6 upregulation in erlotinib-resistant cells line is due to activation of AKT

**Figure 2:**
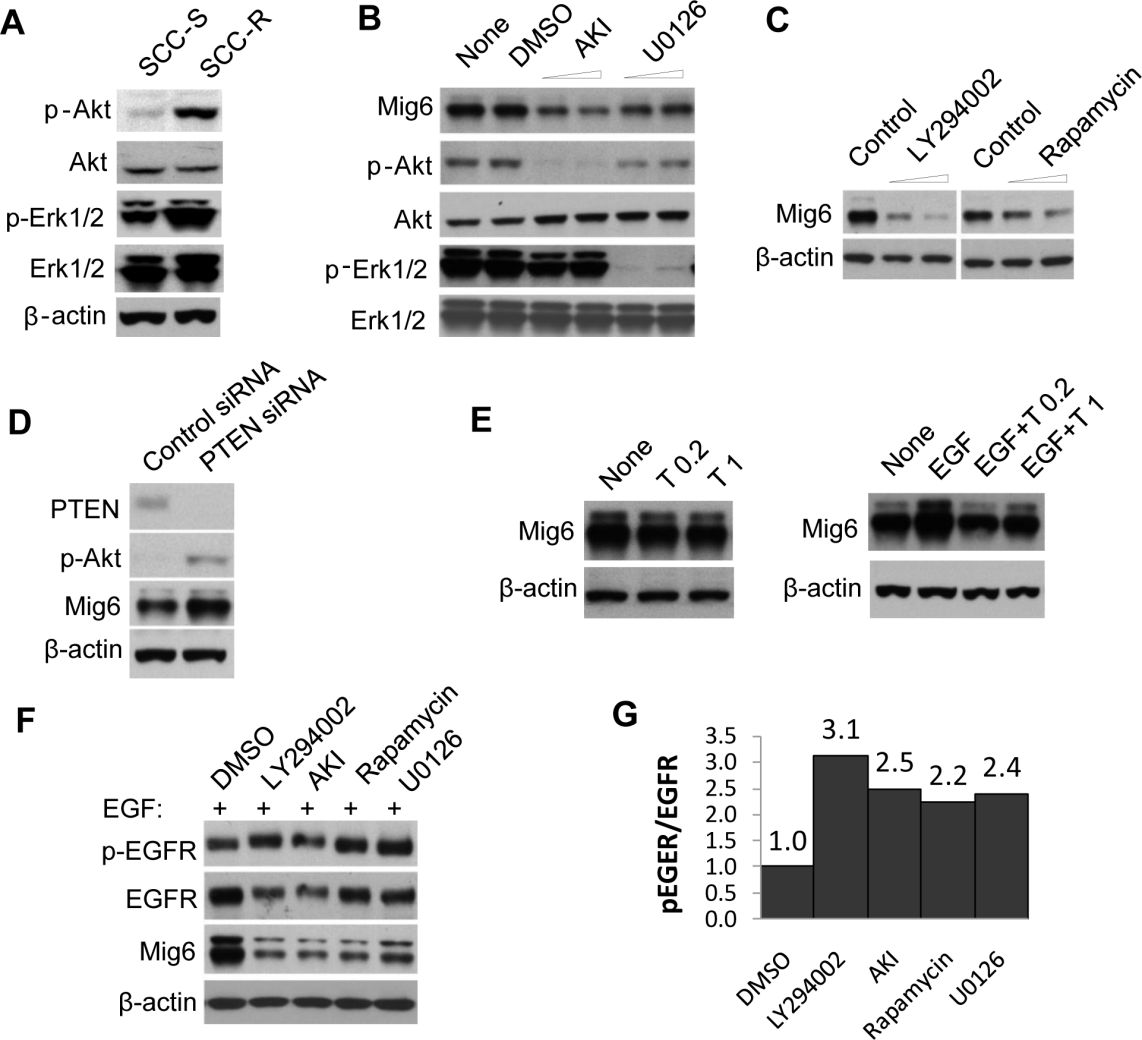
Mig6 expression is upregulated by elevated phospho-AKT in SCC-R cells. **A**) Immunoblot analysis of phospho-AKT, total AKT, and loading control β-actin in SCC-S and SCC-R cells. **B**) SCC-R cells were treated with AKI (AKT1/2 kinase inhibitor, at 5 or 10 µM), U0126 (MEK1/2 inhibitor, at 5 or 10 µM), or DMSO (control) for 24 hrs and subjected to immunoblot analysis with indicated antibodies. **C**) SCC-R cells were treated with LY294002 (PI3K inhibitor, at 5 or 10 µM), rapamycin (mTOR inhibitor, at 1 or 2 µM) or DMSO (control) for 24 hrs and subjected to immunoblot analysis with the indicated antibodies. **D**) SCC-R cells were transfected with either scrambled siRNA or siRNA targeting PTEN for 48 hrs and subjected to immunoblot analysis. **E**) SCC-R cells were treated with 0.2 or 1 µM erlotinib (T0.2, T1, respectively) for 24 hrs, or pretreated with 0.2 or 1 µM erlotinib for 30 min and then co-treated with 10 ng/ml EGF for an additional 24 hrs. Mig6 levels were then evaluated with immunoblot analysis. **F**) SCC-R cells were treated with 10 µM LY294002, 10 µM AKT1/2 kinase inhibitor, 1 µM rapamycin, or 10 µM U0126 for 24 hrs. Cells were then treated with 10 ng/ml EGF for 30 min to induce EGFR phosphorylation and subjected to immunoblot analysis. **G**) Densitometric analysis of phospho-EGFR/total EGFR. DMSO-treated samples were arbitrarily assigned a value of 1 and values of the remaining samples represent fold changes of phospho-EGFR per EGFR molecule. Note that fresh Mig6 antibody recognizes a nonspecific band above the Mig6 protein, which gradually disappears after antibody re-suing or recycling.

EGFR-independent activation of the phosphatidylinositol 3-kinase (PI3K) pathway has frequently been seen in cells that develop resistance and is thought to confer resistance to EGFR TKIs [16], [17]. We also observed that the basal phosphorylation level of AKT was higher in SCC-R cells than their sensitive counterparts (Figure 2A). It has previously been shown that Mig6 is regulated by the MEK/ERK pathway [18] and we did find higher ERK1/2 phosporylation in SCC-R cells (Figure 2A). We sought here to determine whether the PI3K pathway was also involved in regulating the basal expression level of Mig6 in SCC-R cells. Treatment of SCC-R cells with either an AKT1/2 kinase inhibitor (AKI, at 5 and 10 µM) or a MEK inhibitor (U0126, at 5 and 10 µM) decreased expression of Mig6 in association with the specific inhibition of each targeted pathway (Figure 2B). Likewise, treatment of SCC-R cells with the PI3K inhibitor, LY294002 (at 5 and 10 µM), and the mTOR inhibitor, rapamycin (at 1 and 2 µM), also decreased Mig6 expression (Figure 2C). Conversely, direct activation of the PI3K-AKT pathway via RNAi-mediated silencing of PTEN expression resulted in an increase in Mig6 expression (Figure 2D). In keeping with the role of EGFR-independent growth factor receptors in activating PI3K-AKT-mediated upregulation of Mig6, treatment of SCC-R cells with erlotinib (at 0.2 and 1 µM) produced only a slight decrease in basal Mig6 expression (Figure 2E), even though erlotinib could completely abolish EGF-induced Mig6 upregulation (Figure 2E). Furthermore, exposure to each inhibitor (LY294002, AKI, rapamycin, or U0126, at lower dose indicated above) increased the ratio of phospho-EGFR to EGFR (Figure 2F and 2G) upon ligand stimulation, consistent with the role of Mig6 in regulating EGFR activity. These data indicate that upregulation of PI3K-AKT-mTOR by alternative growth factor receptors promotes Mig6-mediated inhibition of EGFR activity, enabling EGFR-independent growth of tumor cells and rendering them insensitive to EGFR-targeted TKIs. Note that fresh Mig6 antibody recognizes a nonspecific band above the Mig6 protein, which gradually disappears after antibody re-using or recycling.

### Mig6/EGFR expression ratio is associated with erlotinib resistance in cancer cell lines of different tissue origins

**Figure 3:**
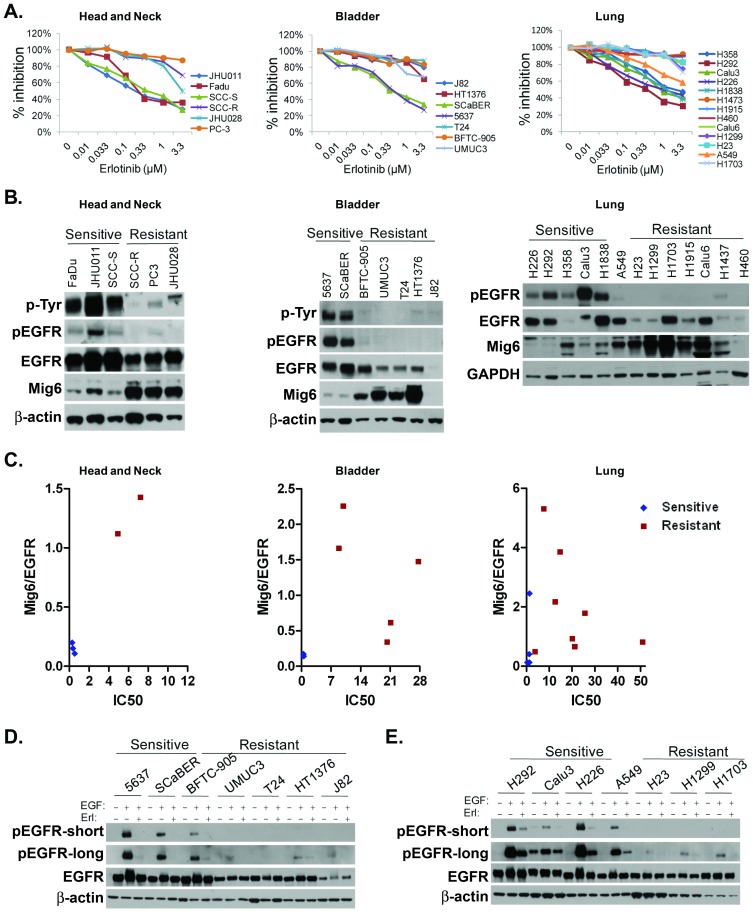
Mig6 upregulation is associated with erlotinib resistance. Head and neck (with PC-3 as prostate), bladder, and lung were treated with indicated doses of erlotinib for 72 hrs and then viable cells were evaluated (**A**). Value was set at 100% for each vehicle-treated cell line. They were evaluated for total and tyrosine phosphorylated forms of EGFR and Mig6 by immunoblot analysis. β-actin or GAPDH were used as internal loading controls (**B**). The exposure density of both EGFR and Mig6 blotted on the same membrane were quantified by densitometry and the values of Mig6/EGFR were plotted against IC50 (**C**). Bladder (**D**) and lung cancer cell lines (**E**) were stripped in serum-free medium overnight and treated with vehicle or 10 ng/ml EGF for 10 min, following pretreatment with vehicle or 0.1 µM erlotinib for 3 hrs. Cells were then subjected to immunoblot analysis for phospho-EGFR and total EGFR. β-actin was used as a loading control. Both shorter (p-EGFR-shorter) and longer (p-EGFR-longer) exposure times for phospho-EGFR are shown to provide more detail for each cell line.

We next investigated Mig6 expression, EGFR expression and EGFR activity in panels of cancer cell lines. At the maximum tolerated and currently used dose of erlotinib (150 mg per day), steady-state serum concentrations range between 0.33 to 2.64 µg/mL with a median of 1.26±0.62 µg/mL or 2.9 µM [19]. Because 90% of erlotinib is bound to serum proteins, the free drug concentration is approximately 0.3 to 1 µM. Therefore, for this study cells were defined as erlotinib-sensitive when significant cell growth inhibition (IC_50_) was observed at a concentration of erlotinib less than or equal to 1 µM, while cells that failed to undergo such growth inhibition were considered erlotinib-resistant. Lung cancer cell line A549 was considered intermediate-resistant based on its erlotinib response curve. Our data indicated that higher Mig6 expression was strongly associated with lower levels of EGFR phosphorylation and erlotinib resistance in 6 of 6 head and neck and prostate cancer cell lines assayed (Figure 3A and B). Similar results were also observed in 17 of 20 bladder and lung cancer cell lines (Figure 3A and B). The exceptions to this pattern (J82-bladder cancer cell line, H1437 and H460-lung cancer cell lines) all showed low levels of Mig6, yet displayed an erlotinib-resistant phenotype. In each of these cases, the cells displayed very low EGFR expression when compared to their erlotinib-sensitive counterparts. Thus, across the cell lines tested, the ratio of Mig6 to EGFR, appeared to be a more reliable predictor of tumor cell response to erlotinib than the absolute expression of either protein alone (Figure 3C).

The association between high Mig6/EGFR ratio and erlotinib resistance suggests that tumor cells that have low EGFR activity will be largely unresponsive to EGFR TKIs. In this situation, the resistance of tumor cells to EGFR inhibition results from the functional irrelevance of EGFR as opposed to the inability of these agents to inhibit basal or ligand-induced EGFR activity. To test this hypothesis, bladder and lung cancer cell lines were exposed to vehicle or erlotinib prior to treatment with EGF. EGF induced heavy EGFR phosphorylation in all sensitive cell lines, while only light phosphorylation was observed in the erlotinib-resistant cell lines tested (Figure 3D). Importantly, erlotinib was able to effectively block ligand-induced EGFR phosphorylation in all cell lines tested, indicating that the ability of erlotinib to block EGFR activation was not impaired even after cells developed resistance to its growth inhibitory effects.

To further investigate the relationship of p-AKT, p-ERK1/2 and Mig6 to the sensitivity of erlotinib, we again blotted the 26 cell line panel and plotted protein expression level against the IC50 of erlotinib (Figure S1). Our data showed that Mig6 expression was associated better with p-AKT than p-ERK1/2, which suggested that p-AKT pathway might be playing bigger role in regulating Mig6. Interestingly, our data also suggested that erlotinib sensitivity was associated better with Mig6 (*P* = 0.0002) than p-AKT (*P* = 0.002).

Since AKT was highly activated in the resistant cells when EGFR activity was low, we next sought to find out whether other growth factor pathways were activated in the resistant cells. We performed p-RTK arrays on parental and acquired resistant HN cell lines (SCC-S and SCC-R), as well as one sensitive (H358) and one resistant (H1703) lung cancer cell line. Our data again confirmed that EGFR family phosphorylation was lower in the resistant cells (SCC-R and H1703) and other RTKs were activated instead, such as PDGFR, FGFR,VEGRR, c-MET, FLT-3 and AXL (Figure S2). These data suggested a kinase switch when cells acquire resistance to erlotinib.

### Knocking down Mig6 *per se* is not sufficient to increase basal EGFR activity and alter erlotinib sensitivity

**Figure 4:**
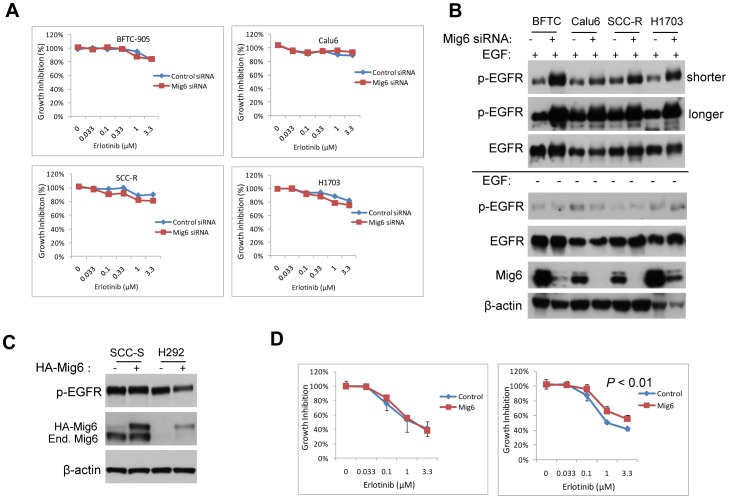
Mig6 knockdown *per se* did not alter cells sensitivity to erlotinib. **A**) Cells were transfected with either control siRNA, or siRNA targeting Mig6 and erlotinib sensitivity assay was performed. **B**) Cells were transfected with either control siRNA, or siRNA targeting Mig6 and immunoblot blots were performed. **C**) Cells were infected with MSCV-HA-Mig6 and immunoblot blots were performed. End. Mig6: endogenous Mig6. **D**) Erlotinib sensitivity was tested in control or HA-Mig6 expressing cells. Data are plotted as mean ± SD and values were set at 100% for untreated controls. * indicates *P*<0.01.

Mig6 knockdown has been previously shown to increase cellular sensitivity to anti-EGFR therapeutic agent such as cetuximab [20]. Unexpectedly, we found that depletion of Mig6 *per se* failed to increase the sensitivity of cells to erlotinib significantly (Figure 4A). These data are in contrast to those reported by Adam L et al, in which Mig6 knockdown reversed resistance to EGFR therapy [20]. Immunoblotting data showed that basal EGFR level was not significantly affected in an unstimulated environment by Mig6 depletion, despite the fact that EGFR phosporylation was strongly enhanced by Mig6 depletion after ligand stimulation (Figure 4B). These data suggested that EGFR activity (cellular dependence on EGFR), rather than the absolute expression level of Mig6, might underlie the response of cancer cells to erlotinib. To confirm this, we next infected SCC-S and H292 cells with a MSCV retrovirus carrying HA-tagged Mig6 and examined EGFR phosporylation and erlotinib. Infection with MSCV and selection with blasticidine for 3 days resulted in expression of HA-Mig6 in H292 cells which lacks endogenous Mig6 (Figure 4C). In SCC-S cells, the expression of HA-Mig6 was of similar level as that of the endogenous Mig6 (End. Mig6, Figure 4C). Interestingly, introduction of Mig6 to H292 cells significantly increased resistance to erlotinib when concomitantly decreased basal EGFR phosporylation was seen (Figure 4C and 4D, *P*<0.01). However, it did not affect sensitivity to erlotinib in SCC-S cells where EGFR phosporylation was not affected (Figure 4C and 4D). Taken together, our data suggested that cellular dependence on EGFR, which can be predicted by basal Mig6/EGFR ratio, underlie the response of cancer cells to erlotinib rather than the absolute expression level of Mig6. This was further supported by our observation that Mig6/EGFR demonstrated a high degree of accuracy as the predictor of EGFR activity in a large panel of head and neck, bladder and lung cancer cell lines examined. In addition, in review of data from a published report, the relative expression of Mig6 and EGFR also correlates well with basal EGFR activity in a panel of breast cancer cells examined [15].

To understand whether Mig6 knockdown in combination with p-AKT inhibition sensitize cells to erlotinib, we knocked down Mig6 and treated cells with AKT inhibitor. We found that AKT pathway inhibition could be detrimental to the resistant cells over the period of a few days. However, co-treatment with low dose of AKT inhibitor (5 µM) did sensitize cells to erlotinib in H1703 cells (Figure S3).

### The expression of Mig6 and EGFR in predicting erlotinib sensitivity in directly xenografted human lung and pancreatic tumors

**Figure 5:**
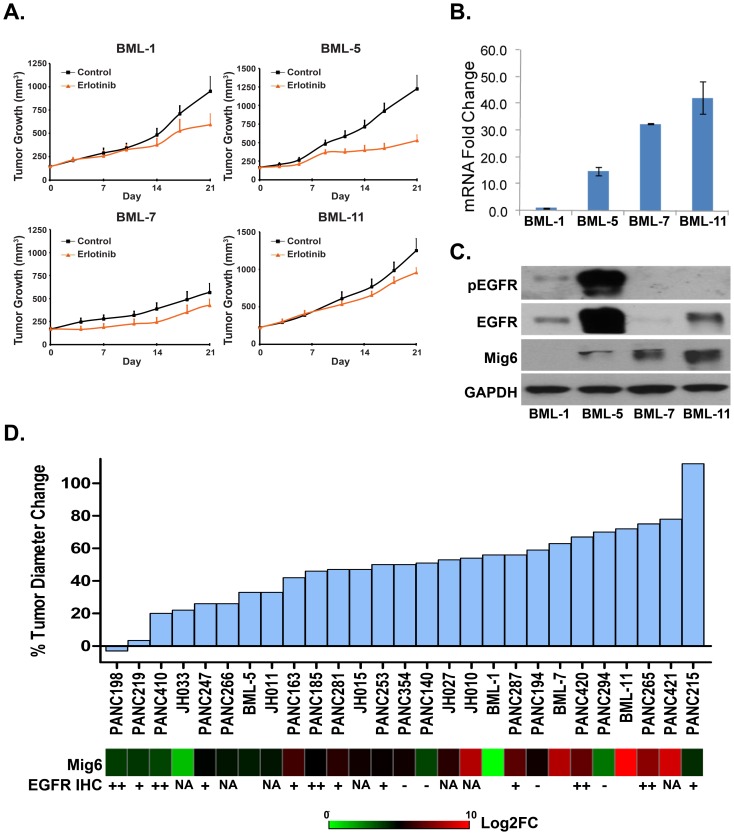
Mig6 expression correlated with erlotinib response in directly xenografted low passage lung and pancreatic tumors. **A**) Effect of erlotinib on growth of directly xenografted lung tumors. Data are plotted as mean ± SEM. **B**) Real-time PCR of Mig6 on directly xenografted tumors. Data are plotted as mean ± SD after normalization with GAPDH. **C**) Immunoblot analysis of protein lysates of lung xenografts. **D**) Efficiency of erlotinib in inhibiting growth of tumor xenografts was displayed from most sensitive (left) to most resistant (right) as a bar graph. Relative expression of Mig6 in each tumor xenograft is displayed underneath the tumor growth inhibition bar as a heatmap. FC: fold change. Scale used was Log_2_FC.

To investigate whether our observations with tumor cell lines could be validated in tumor samples from patients, we analyzed directly xenografted low passage human tumors that have been shown to retain the key features of the original tumor, including drug sensitivity, and that accurately represent the heterogeneity of the disease [21]. We obtained 4 human NSCLCs, and 18 pancreatic tumors that were directly xenografted into nude mice [22]. No erlotinib-sensitizing mutations in EGFR were detected in any of these tumors. We initially tested the response of the 4 patient-derived lung xenografts (BML-1, BML-5, BML-7 and BML-11) to erlotinib. Among them, BML-5 showed a better response to erlotinib than the other 3 tumors (Figure 5A). Analysis of Mig6 expression in tumor xenografts showed that BML-1 and BML-5 expressed less Mig6 than BML-7 and BML-11 (Figure 5B and C). In addition, BML-5 expressed higher total EGFR as well as higher basal EGFR phosphorylation than the other tumors (Figure 5B and C).

We next characterized and plotted erlotinib responsiveness of 18 directly xenografted pancreatic tumors. Tumor growth inhibition data are displayed with the most sensitive tumors on the far left and the most resistant on the far right (Fig. 5D). Tumor characteristics, including KRAS mutation status as well as EGFR expression and phosphorylation levels, have been reported previously [22], [23]. No EGFR sensitizing-mutations were found in any of these tumors and there was no correlation of KRAS mutation with erlotinib response in pancreatic tumors [22], [23]. EGFR negative tumors tended to cluster on the right side of the map, indicating that they were more resistant to erlotinib. However, in EGFR-positive tumors we saw little association between erlotinib sensitivity and EGFR expression (Figure 5D). Instead, we found that in these pancreatic tumors, as Mig6 expression increased, tumors exhibited a more erlotinib-resistant phenotype. For example, the erlotinib-resistant tumor PANC420 expressed markedly higher Mig6 than the erlotinib-sensitive tumor PANC410, even though they expressed comparable amounts of EGFR protein [22], [23]. In keeping with their Mig6 expression status, PANC410 displayed heavy EGFR phosphorylation whereas PANC420 harbored no detectable EGFR phosphorylation [22], [23]. Interestingly, in the 3 erlotinib-resistant pancreatic tumors studied that displayed significantly lower Mig6 expression (PANC140, 294, and 215), IHC labeling revealed that 2 of these 3 xenograft lines did not express EGFR [22].

### Mig6/EGFR expression ratio correlates with the response of patients to gefitinib

**Figure 6:**
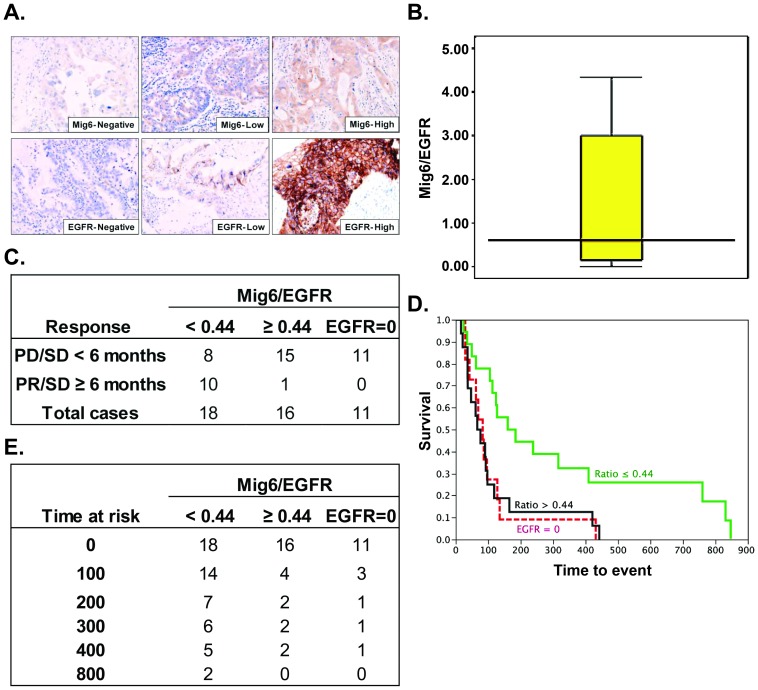
Mig6/EGFR ratio correlates with the response of patients to gefitinib. **A**) Selected pictures of IHC staining against Mig6 and EGFR. **B**) Box plot of Mig6/EGFR ratio distribution. **C**) The response of patients to gefitinib treatment. PD, progressive disease; SD, stable disease; PR, partial response. **D**) Kaplan-Meier survival curves showed that patients with low Mig6/EGFR ratio survived significantly longer than the high ratio patients and EGFR negative patients (Log-Rank test *P* = 0.0112). **E**) The number of patients as risk at the time point of 0, 100, 200, 400 and 800 days was displayed in the table.

To investigate whether relative levels of Mig6 and EGFR expression correlate with the clinical drug response to anti-EGFR TKIs, we examined Mig6 and EGFR expression immunohistochemically and in blinded fashion on tissues from a cohort of lung cancer patients who had previously been treated prospectively with gefitinib alone (Figure 6A). Mig6 cytoplasmic expression and EGFR membranous expression were analyzed in tumor cells using a score calculated intensity (0–3+) multiplied by extension of expression (0–100%; range 0–300). Expression ratios were calculated as Mig6 expression/EGFR expression (ratios ranged from 0 to 4.33, figure 6B). We grouped the patients with positive EGFR (>0) staining in low or high Mig6/EGFR ratio groups using the number close to median (0.44) as the cutoff. Our data showed that the 2 patients who had partial response (PR) were exclusively in the low ratio group, with ratios of 0 and 0.14 (Table S1). In addition, patients with lower Mig6/EGFR ratio have significant better outcome than the rest of the patients (Fisher exact test, *P* = 0.002, figure 6C). 10/18 (55.6%) patients have combined PR and stable disease (SD) ≥6 months in the low ratio group, but this number is only 1/16 (6.3%) in the high ratio group (P<0.001, figure 6C). Kaplan-Meier survival curves showed that patients with a low Mig6/EGFR ratio survived statistically significantly longer than the high ratio patients and EGFR negative patients (Figure 6D, Log-Rank test *P* = 0.01). The number of patients at risk at different time points was shown in Figure 6E. The median progression-free survival (PFS) was 96 days for the entire cohort, 71 days for high ratio group, and 83 days for EGFR negative group. However, the median PFS in low ratio group was 172 days, approximately 100 days longer than patients in either the high or EGFR negative groups. These data suggest that patients whose tumors express lower Mig6/EGFR ratio were much more responsive to gefitinib treatment. The statistical significance of this comparison was sensitive to the choice of cutpoint for the ratio, so the optimal ratio should be tested in a prospective trial.

## Discussion

Studies have suggested a weak association between EGFR protein expression levels and responsiveness to EGFR TKIs [2], [8], [9], [10]. Although the erlotinib-sensitive tumors studied here generally displayed high EGFR levels, our data suggested that it was the activity of EGFR rather than its level of expression most accurately predicted drug response. In supporting of these findings, activation of the EGFR pathway has previously been reported to be the only reliable predictive factor of erlotinib responsiveness in pancreatic cancer patients [22], [23]. In addition, when sensitive cancer cells are transformed to a lower phospho-EGFR phenotype, as is seen in an induced EMT-like transition, erlotinib resistance occurs [24]. Our data also suggest that the relative expression of the ERBB family negative regulator Mig6 and EGFR, strongly correlated with EGFR activity in EGFR positive tumors. Cancer cells with EGFR overexpression could be erlotinib-resistant due to reduced dependence on EGFR signaling as predicted by higher Mig6 expression levels. Neoplastic cells with a low Mig6/EGFR ratio may exhibit active EGFR signaling and sensitivity to EGFR TKIs, while those with a high Mig6/EGFR ratio frequently display reduced EGFR activity and resistance to EGFR TKIs.

In cell lines that acquired resistance to erlotinib we found that Mig6 upregulation was driven by markedly elevated basal PI3K-AKT activity. Since Mig6 functions as a negative regulator of EGFR activity, PI3K-AKT-mediated upregulation of Mig6 could negatively regulate signal input from EGFR once a cancer cell senses adequate growth and survival signals from alternative sources. This change would allow cells to shift their cellular phenotype towards a less EGFR-dependent state. We have observed upregulation of multiple growth factor receptors and their ligands in the acquired resistant cells. Similar to our observation, a recent report on therapeutic resistance to the anti-ERBB2 agent trastuzumab demonstrated that all of the acquired resistant cell lines displayed reduced ErbB2 signaling with concomitant enhanced alternative RTKs signaling [25].

Despite the fact that Mig6/EGFR was highly associated with EGFR activity in cancer cell lines of multiple tissue types, depleting Mig6 *per se* in these cells failed to alter basal EGFR activity and the response to erlotinib in an unstimulated environment. However, Mig6 reduction drastically increased the activity of EGFR following ligand stimulation. These results might be explained by the recent data which showed that Mig6 inhibits EGFR via a two-tiered mechanism which involves receptor degradation and trafficking in addition to kinase suppression [26], [27]. In contrast to our results, a recent study demonstrated that depleting Mig6 *per se* in cetuximab-resistant bladder cell lines increased their sensitivity to the drug [20]. It is not clear whether the discrepancy is due to cell type specificity, but our results suggest that EGFR activity, rather than the absolute expression level of Mig6, underlies the response of cancer cells to anti-EGFR agents. Nevertheless, others have previously demonstrated that mouse embryo fibroblasts (MEF) from *Errfi1^−/−^* mice, driven by aberrantly active EGFR, proliferate more rapidly than those from the *Errfi1^+/+^* mice [28], while carcinogen-generated tumors that develop in Mig6 knockout mice are highly sensitive to gefinitib. Tumors in *Errfi1^−/−^* mice regressed more than 50% in 1 week following initiation of gefitinib treatment, whereas those in control *Errfi1^+/+^* mice did not respond to gefitinib [15]. In addition, Mig6/EGFR as a predictor of EGFR activity or erlotinib resistance demonstrated a high degree of accuracy in head and neck, bladder and lung cancer cell lines, primary xenografts, and patient samples. Our work identifies the potential clinical utility of the Mig6/EGFR ratio as a biomarker. The increased response rate and progression free survival observed here in patients with lung cancer whose tumors demonstrated a low Mig6/EGFR ratio are dramatic. The first IDEAL trial in NSCLC randomizing patients to gefinitib or placebo showed an overall difference of PFS of only 7 days [29], as compared to the median survival difference of nearly 100 days seen here. This finding further highlights the need to identify those patients most likely to respond to and benefit from therapy when treatment efficacy is evaluated. As an approach to personalized therapy, the expression levels of both EGFR and Mig6 could be examined in tumor cells, and the ratio of the 2 molecules could be used to select patients who are likely to benefit from anti-EGFR therapy. Subsequent increase in this ratio might indicate the development of drug resistance. Since Mig6 played a consistent role across multiple tumor types, the Mig6/EGFR ratio may be further clinically tested as a novel biomarker for predicting TKI response (and perhaps antibodies to EGFR as well) in diverse epithelial cancers. These findings provide a scientific foundation for validating the predictive accuracy of biomarkers gleaned from observations in primary human tumorgrafts in prospective clinical trials. Lastly, our work underscores the role of negative regulators of receptor RTKs in cellular utilization of these receptors and should be taken into consideration for drug response evaluation of any molecular targeted therapies to other RTKs.

## Materials and Methods

### Compounds and reagents

Erlotinib (OSI-774, Tarceva) was purchased from Johns Hopkins University Hospital Pharmacy. LY294002 and U0126 were obtained from Cell Signaling Technology, Inc. (Beverly, MA). EGF was purchased from BD Pharmingen (San Diego, CA). All other chemicals were purchased from Sigma (St. Louis, MO), except where otherwise indicated. All chemicals and growth factors were dissolved in recommended vehicle as instructed by the manufacturers.

### Cell lines

The human NSCLC cell lines (H226, H292, H358, H1838, A549, Calu6, H460, H1703, H1915, H1299, Calu3, H1437, and H23), human bladder cancer cell lines (5637, SCaBER, UMUC-3, T24, HT-1376 and J82), and human head and neck squamous cell carcinoma (HNSCC) cell line FaDu were obtained from American Type Culture Collection (ATCC). BFTC-905 was obtained from German Collection of Microorganisms and Cell Cultures (Braunschweig, Germany). The majority of the cell lines were freshly ordered and used within 6 month of order date. Old cell lines were fingerprinted to ensure the authority of the cells and our laboratory periodically monitors mycoplasma and decontaminates the cells.

### Establishment of acquired resistance to erlotinib

Drug resistant cell lines were generated via a process of slowly escalating exposure to erlotinib, as reported previously [30]. SCC-S is used to designate the parental UM-SCC1 cells exposed to DMSO, and SCC-R refers to the erlotinib resistant clone.

### siRNA transfection

Mig6 siRNA was synthesized and purchased from Invitrogen (Carlsbad, CA) according to published sequences [15]. PTEN siRNA was obtained from Cell Signaling Technology, Inc. (Beverly, MA), and EGFR siRNA was purchased from Santa Cruz Biotech (Santa Cruz, CA). Cells were plated in either 6-well or 96-well plates and transfected with the indicated siRNA using RNAiMAX transfection reagent (Invitrogen, Carlsbad, CA) according to the manufacturer's instructions. Cells were subjected to immunoblot blot analysis or viability assay 72 hrs post-transfection, unless otherwise stated.

### HA-Mig6 retrovirus infection

The Murine Stem Cell Virus (MSCV-blast) retrovirus carrying HA tagged human full-length Mig6 was packaged and produced in 293 cells system per manufacturer's instructions (Clontech, CA). The vector was a generous gift from Dr. R. DePinho. Briefly, MSCV-Mig6 vector were co-transfected with V-SVG into 293GP cells. 60 hours after tranfection, media containing virus were collected and filtered through 0.45 µM filter. SCC-S and H292 were infected with the virus for 8 hrs, recovered overnight, and infected again for additional 8 hrs. 24 hr after infection, cells were treated with blasticidine for additional 3 days before cells were used for drug sensitivity assay.

### Antibodies and immunoblot analysis and immunoprecipitation analysis

Antibodies against EGFR, phospho-tyrosine (P-Tyr-100), phospho-EGFR (Tyr1068), phospho-HER2/ErbB2 (Tyr1248), AKT, phospho-AKT (Ser473), p44/42 MAPK (Erk1/2), phospho-p44/42 MAPK (Erk1/2) (Thr202/Tyr204), and PTEN were obtained from Cell Signaling Technology (Beverly, MA). Polyclonal anti-Mig6 antibody was a generous gift from Dr. Ferby [15]. When appropriate, cells were cultured in serum free medium overnight, pretreated with the indicated inhibitors for 3 hrs or 24 hrs, and the treated with 10 ng/ml EGF for 10 or 30 min and immunoblot and immunoprecipitation analysis were performed as previously described [31].

### Reverse transcription (RT) and real-time PCR

RNA was extracted using Trizol (Invitrogen, Carlsbad, CA) followed by RNeasy kit cleanup (Qiagen, Valencia, CA). RNA was reverse transcribed to cDNA using Superscript III (Invitrogen) which was then used as a template for real-time PCR. Gene products were amplified using iTaq SYBR green Supermix with Rox dye (Bio-Rad Laboratories, Hercules, CA). All reactions were performed in triplicate, with water controls, and relative quantity was calculated after normalizing to GAPDH expression. The primer sequences of Mig6 were 5′-CTACTGGAGCAGTCGCAGTG -3′ (forward) and 5′-CCTCTTCATGTGGTCCCAAG -3′ (reverse), and primer sequences for GAPDH were: 5′-CAACTACATGGTTTACATGTTC-3′ (forward) and 5′-GCCAGTGGACTCCACGAC (reverse). Expression of Mig6 mRNA relative to GAPDH was calculated based on the threshold cycle (Ct) as 2^−Δ(ΔCt)^, where Δ(ΔCt) = ΔCt_Mig6_ – ΔCt_GAPDH_.

### Cell viability and drug sensitivity assay

Cells were plated at a density of 3000/well in 96-well plates. The following day, cells were treated with 0, 0.01, 0.033, 0.1, 0.33, 1, or 3.3 µM erlotinib for an additional 72 hrs. Cell viability was subsequently assayed using Calcein AM (Invitrogen). Fluorescence signals generated as a result of Calcein AM cleavage by viable cells were read by a Molecular Devices plate reader (Sunnyvale, CA) using an excitation frequency of 480 nm, and an emission frequency of 535 nm. For AKT inhibition experiment, cells were depleted for Mig6 using siRNA and RNAiMAX transfection reagent (Invitrogen, Carlsbad, CA). Nonspecific siRNA was used as a control. 24 hours after transfection cells were pretreated with 5 µM AKT1/2 inhibitor (Sigma Aldrich, St. Louis, MO) for 6 hours and then treated with indicated concentrations of Erlotinib for additional 72 hours.

### Human Phospho-Receptor Tyrosine Kinase (p-RTK) Array

Human p-RTK array (R&D systems, Minneapolis, MN) was used for the parallel determination of the relative level of tyrosine phosphorylation of 49 different human RTKs. Briefly, capture and control antibodies have been spotted in duplicate on nitrocellulose membranes. After blocking, lysates from two pairs of resistant and sensitive cells were incubated with the Human Phospho-RTK Array overnight. After binding the extracellular domain of RTKs, unbound material is washed away and a pan anti-phospho-tyrosine antibody conjugated to horseradish peroxidase (HRP) is then used to detect phosphorylated tyrosines on activated receptors by chemiluminescence.

### Xenograft generation in mice and erlotinib treatment

The xenografts were generated and erlotinib treatment was performed as published previously [22], [23]. Relative tumor growth inhibition (TGI) was calculated as the relative tumor growth of treated mice divided by relative tumor growth of control mice (T/C). The animals were maintained in accordance to guidelines of the American Association of Laboratory Animal Care and the research protocol was approved by the Johns Hopkins University Animal Use and Care Committee.

### Immunohistochemistry (IHC) staining for Mig6 and EGFR

IHC were performed using an automated stainer (Dako Inc., Carpinteria, CA). Anti-Mig6 antibody was purchased from Sigma, and anti- EGFR were ordered from Dako Inc. (Carpinteria, CA). Tissue processing, deparaffinization, antigen retrieval and IHC staining were performed as directed by the manufacturer. Briefly, staining was performed by serially incubating tissue sections in Methanol/3% H_2_O_2_ (15 min), PBS, serum free protein (block) (7 min), rabbit anti-Mig6 or EGFR antibody (90 min at 22°C), PBS (rinse), biotinylated secondary antibody (DAKO) (30 min at 22°C), PBS, streptavidin-HRP (DAKO) (30 min at 22°C), and PBS. Staining was visualized with 3,3′-diaminobenzidine (DAB) tetrahydrochloride (Zymed, Carlsbad, CA).

### Patient selection

Formalin-fixed, paraffin-embedded (FFPE) tumor tissue samples were obtained from patients with advanced non-small cell lung carcinoma treated with gefitinib or erlotinib at The University of Texas M. D. Anderson Cancer Center between May 1999 and December 2004 [32]. There were 45 samples available which were all included in this study. All tumor specimens were histologically classified according to the WHO classification for lung cancer by an experienced thoracic pathologist (I.I.W.) [33]. Clinical response was graded according to the Response Evaluation Criteria in Solid Tumors [32], [34].

### Statistical analysis

Student *t*-tests were used for statistical analysis between two groups. All *P* values are based on two-sided. The significance level was defined as 0.05. Survival analysis was performed using Kaplan-Meier model and significance was determined using a two-sided log-rank test. All statistical analyses were performed using SPSS. IC50 was generated using GraphPad Prism software (La Jolla, CA).

## Supporting Information

Figure S1 **The relationship of p-AKT, p-ERK1/2 and Mig6 to the sensitivity of erlotinib.** A) Immunoblot analysis of phospho-AKT, total AKT, phospho-ERK1/2, total ERK1/2 and Mig6 in indicated cancer cell lines. B) The expression level of each molecule was plotted against IC50 of corresponding cell line.
(TIF)

Figure S2 **Phospho-receptor tyrosine kinase (pRTK) arrays were performed on two sensitive (SCC-S and H358) and two resistant cell lines (SCC-R and H1703).** EGFR family members, as well as upregulated RTKs in the resistant cell lines were highlighted in boxes. Note that there were artifact spots on the SCC-S membrane which were not seen in all other three membranes.
(TIF)

Figure S3 **H1703 cells were transfected with either control or Mig6 siRNA and AKT inhibitor was given 6 hrs before the treatment of indicated concentration of erlotinib for additional 72 hrs.** Erlotinib at dose 0 was set as 100% and percentage of survival was determined at indicated erlotinib treatment dosage.
(TIF)

Table S1 **Summary of the clinical and pathological information of 45 patients with advanced non-small cell lung carcinoma included in this study.**
(DOC)
