# Supplementary figures and images for: The Relative Expression of Mig6 and EGFR Is Associated with Resistance to EGFR Kinase Inhibitors
Source: PLoS One. 2013 Jul 31;8(7):e68966. doi: 10.1371/journal.pone.0068966 (PMC3729565; doi:10.1371/journal.pone.0068966)

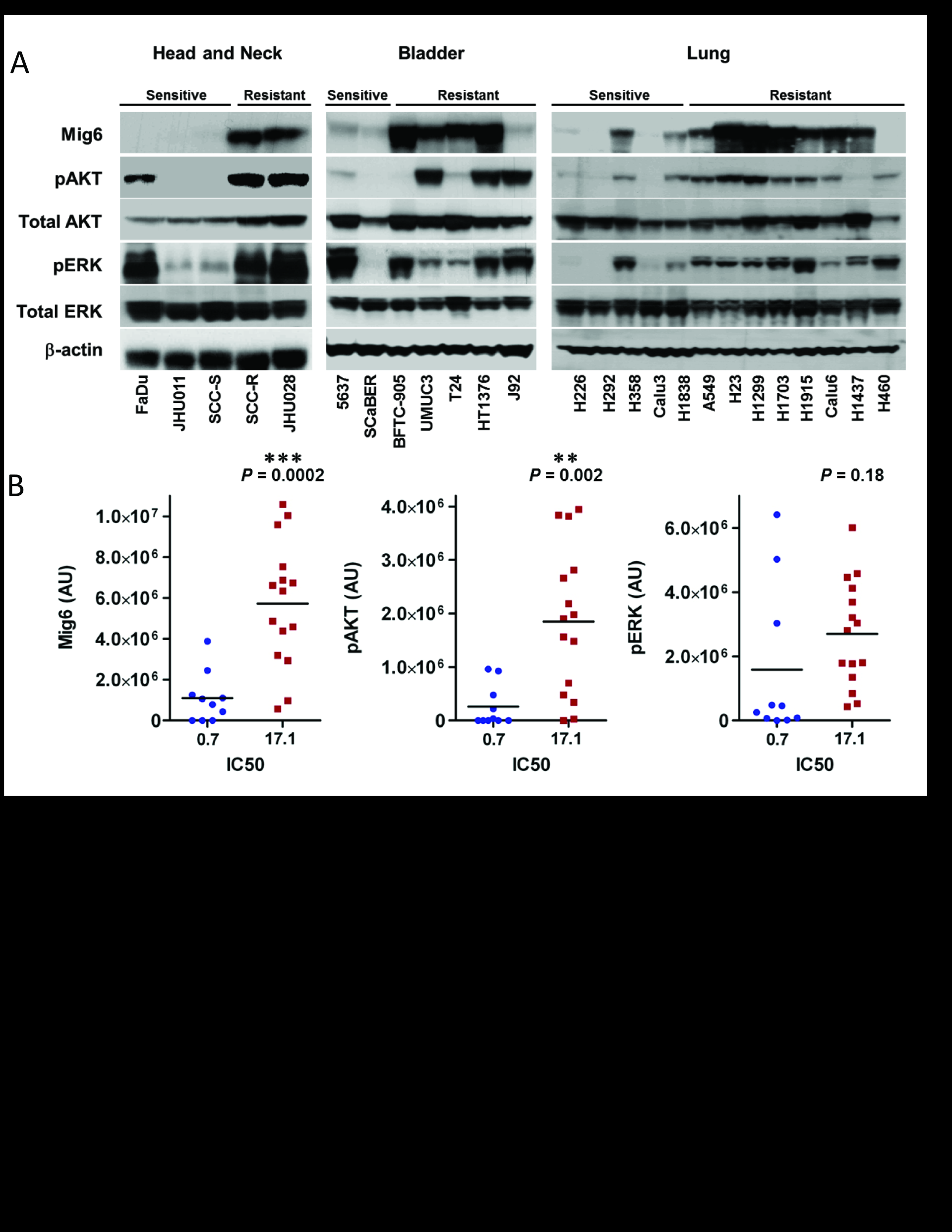

Supplement: Figure S1 — The relationship of p-AKT, p-ERK1/2 and Mig6 to the sensitivity of erlotinib. A) Immunoblot analysis of phospho-AKT, total AKT, phospho-ERK1/2, total ERK1/2 and Mig6 in indicated cancer cell lines. B) The expression level of each molecule was plotted against IC50 of corresponding cell line. (TIF) [file pone.0068966.s001.tif]

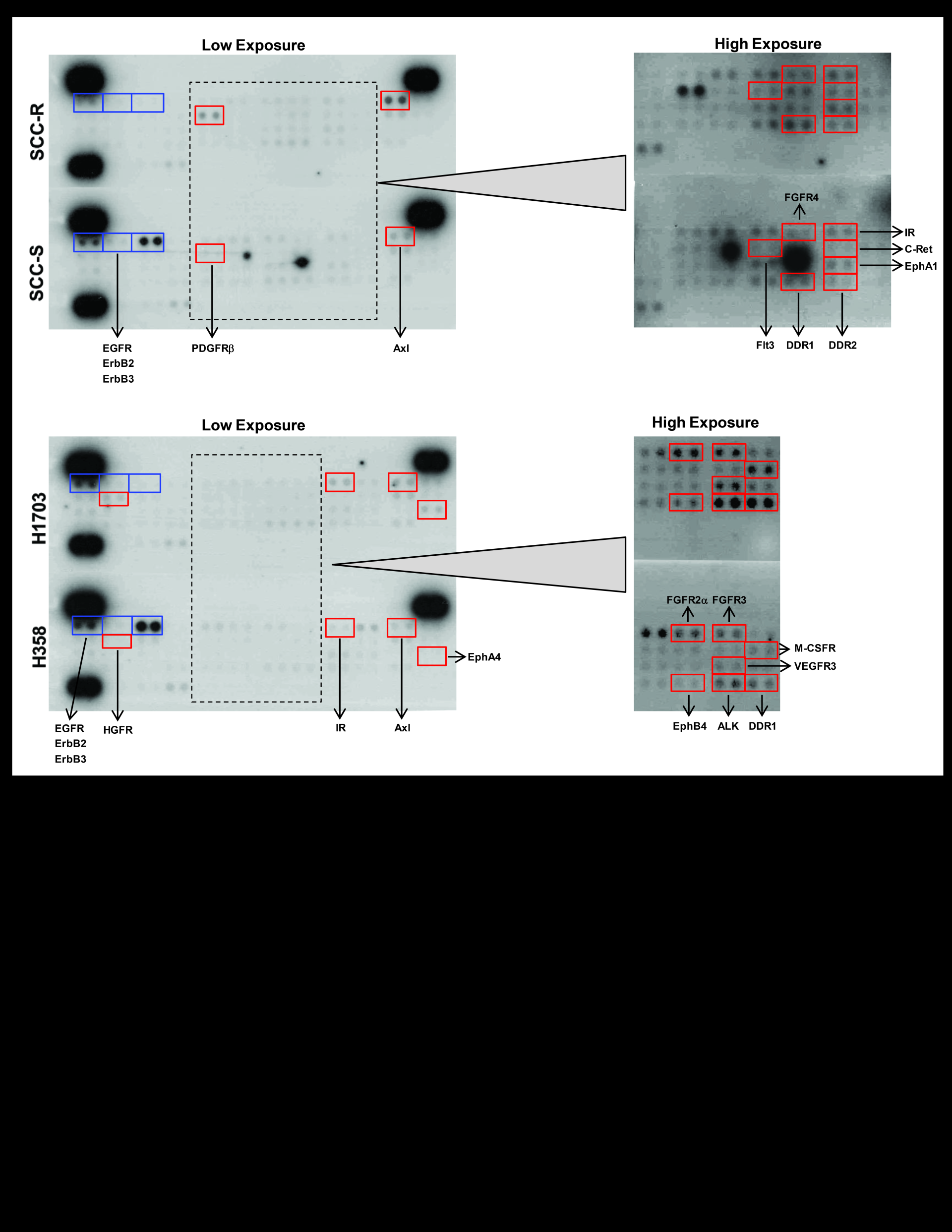

Supplement: Figure S2 — Phospho-receptor tyrosine kinase (pRTK) arrays were performed on two sensitive (SCC-S and H358) and two resistant cell lines (SCC-R and H1703). EGFR family members, as well as upregulated RTKs in the resistant cell lines were highlighted in boxes. Note that there were artifact spots on the SCC-S membrane which were not seen in all other three membranes. (TIF) [file pone.0068966.s002.tif]

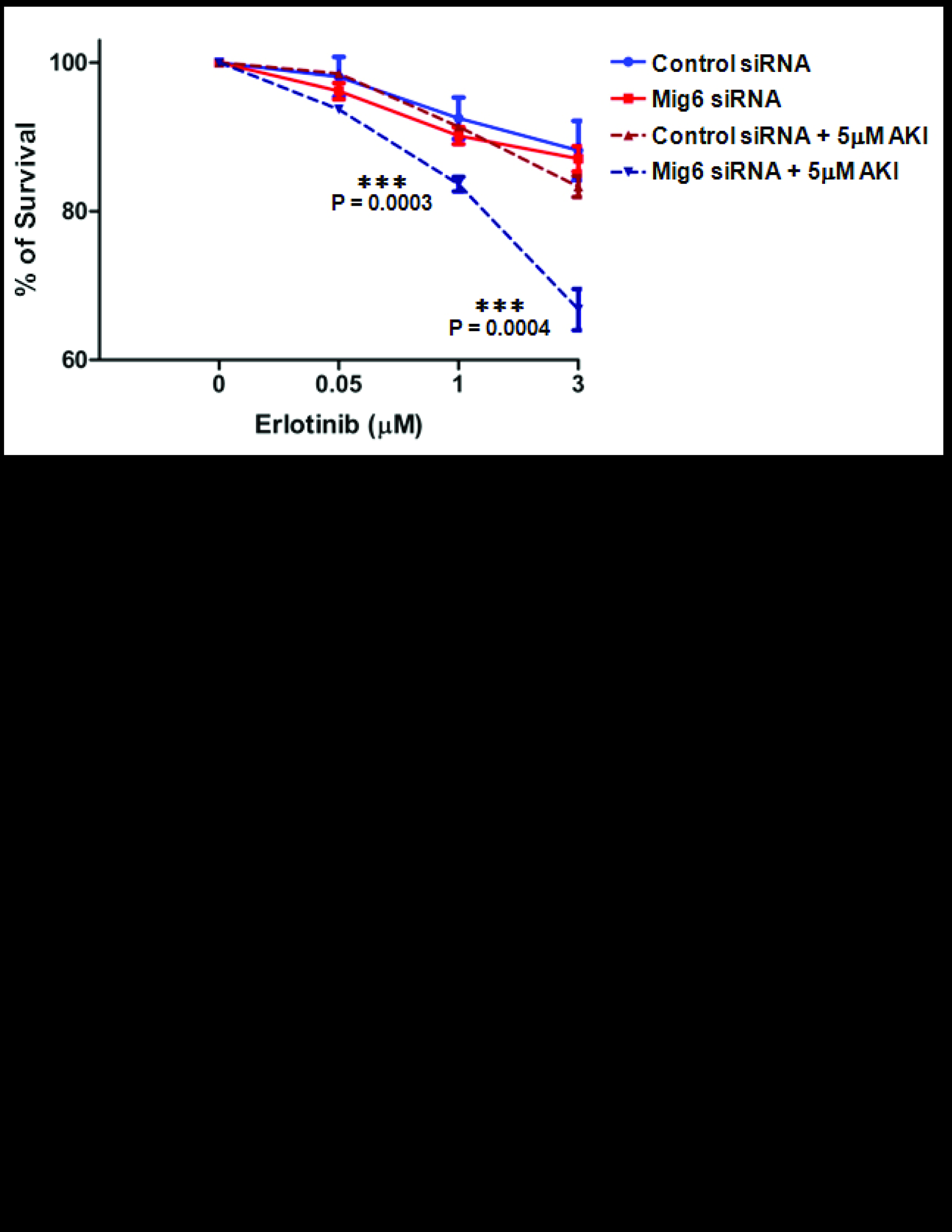

Supplement: Figure S3 — H1703 cells were transfected with either control or Mig6 siRNA and AKT inhibitor was given 6 hrs before the treatment of indicated concentration of erlotinib for additional 72 hrs. Erlotinib at dose 0 was set as 100% and percentage of survival was determined at indicated erlotinib treatment dosage. (TIF) [file pone.0068966.s003.tif]
