# Supplementary material for: The Relative Expression of Mig6 and EGFR Is Associated with Resistance to EGFR Kinase Inhibitors
Source: PLoS One. 2013 Jul 31;8(7):e68966. doi: 10.1371/journal.pone.0068966 (PMC3729565; doi:10.1371/journal.pone.0068966)
Supplement: Table S1 — Summary of the clinical and pathological information of 45 patients with advanced non-small cell lung carcinoma included in this study. (DOC) [file pone.0068966.s004.doc]

**Table S1.** Summary of the clinical and pathological information of 45 patients with advanced non-small cell lung carcinoma included in this study.

|  | **Mig6/EGFR** | | **EGFR=0** |
| --- | --- | --- | --- |
| **Covariate** | **< 0.44** | **≥0.44** |  |
|  | **(n=18)** | **(n=16)** | **(n=11)** |
|  | 57.4 | 61.9 | 59.6 |
| Age, mean, years |  |  |  |
|  |  |  |  |
| Sex |  |  |  |
| Female (n=25) | 7 | 10 | 8 |
| Male (n=20) | 11 | 6 | 3 |
|  |  |  |  |
| Race |  |  |  |
| Asian (n=4) | 0 | 2 | 2 |
| Caucasian (n=34) | 14 | 13 | 7 |
| Other (n=7) | 4 | 3 | 4 |
|  |  |  |  |
| Smoking Status |  |  |  |
| Never (n=11) | 1 | 4 | 6 |
| Former (n=19) | 10 | 7 | 2 |
| Current (n=15) | 7 | 5 | 3 |
|  |  |  |  |
| Histology |  |  |  |
| Adenocarcinoma (n=31) | 2 | 11 | 18 |
| Squamous cell carcinoma (n=10) | 5 | 1 | 4 |
| Large cell carcinoma (n=1) | 0 | 1 | 0 |
| Adenosquamous carcinoma (n=1) | 1 | 0 | 0 |
| NSCLC (n=2) | 2 | 0 | 0 |
|  |  |  |  |
| Disease progression |  |  |  |
| Progressive disease (n=26) | 5 | 12 | 9 |
| Stable disease < 6 mo (n=8) | 3  8 | 3  1 | 2  0 |
| Stable disease ≥ 6 mo (n=9) |  |  |  |
| Partial response (n=2) | 2 | 0 | 0 |
|  |  |  |  |
|  |  |  |  |
|  |  |  |  |
|  |  |  |  |
